# Cardiac magnetic resonance myocardial feature tracking detects quantitative wall motion during dobutamine stress

**DOI:** 10.1186/1532-429X-14-S1-P14

**Published:** 2012-02-01

**Authors:** Andreas Schuster, Shelby Kutty, Asif Padiyath, Victoria Parish, Paul Gribben, David A Danford, Marcus R Makowski, Boris Bigalke, Philipp B Beerbaum, Eike Nagel

**Affiliations:** 1 Imaging Sciences and Biomedical Engineering, KCL, London, UK; 2 Joint Division of Pediatric Cardiology, University of Nebraska/Creighton University, Children’s Hospital and Medical Center, Omaha, NE, USA; 3 Department of Paediatric Cardiology, Evelina Children’s Hospital, Guy's and St. Thomas' NHS Foundation Trust, London, UK; 4 Department of Radiology, Charite, Berlin, Germany

## Summary

We sought to determine the feasibility and reproducibility of cardiac magnetic resonance (CMR) myocardial feature tracking (FT) for quantitative wall motion assessment during intermediate dose dobutamine stress magnetic resonance (DSMR) imaging.

## Background

DSMR imaging is an established tool to assess hibernating myocardium and ischemia. Analysis is typically based on visual assessment with considerable operator dependency. CMR-FT is a recently introduced technique for tissue voxel motion tracking on standard steady-state free precession (SSFP) images to derive circumferential and radial myocardial mechanics.

## Methods

10 healthy subjects were studied at 1.5 Tesla. Myocardial strain parameters were derived from SSFP cine images using dedicated CMR-FT software (Diogenes MRI prototype, Tomtec, Germany). Right ventricular (RV) and left ventricular (LV) longitudinal strain (EllRV and EllLV) and LV long-axis radial strain (ErrLAX) were derived from a 4-chamber view at rest. LV short-axis circumferential strain (EccSAX) and ErrSAX, LV ejection fraction (EF) and volumes were analyzed at rest and during dobutamine stress (10 and 20 μg * kg-1* min-1).

## Results

In all volunteers strain parameters could be derived from the SSFP images at rest and stress. EccSAX values showed significantly increased contraction with DSMR (rest: -24.1±6.7; 10 μg: -32.7±11.4; 20 μg: -39.2±15.2, p<0.05). ErrSAX increased significantly with dobutamine (rest: 19.6±14.6; 10 μg: 31.8±20.9; 20 μg: 42.4±25.5, p<0.05). In parallel with these changes, EF increased significantly with dobutamine (rest: 56.9±4.4%; 10 μg: 70.7±8.1; 20 μg: 76.8±4.6, p<0.05). Observer variability was best for LV circumferential strain (EccSAX ) and worst for RV longitudinal strain (EllRV) as determined by 95% confidence intervals of the difference.

## Conclusions

CMR-FT reliably detects quantitative wall motion and strain derived from SSFP cine imaging that corresponds to inotropic stimulation. The current implementation may need improvement to reduce observer-induced variance. Within a given CMR lab, this novel technique holds promise of easy and fast quantification of wall mechanics and strain.

## Funding

AS receives grant support from the British Heart Foundation (BHF) (RE/08/003 and FS/10/029/28253) and the Biomedical Research Centre (BRC-CTF 196). SK receives grant support from the American College of Cardiology Foundation, the Edna Ittner Pediatric Foundation, and the Children’s Hospital and Medical Center Foundation.

**Figure 1:**
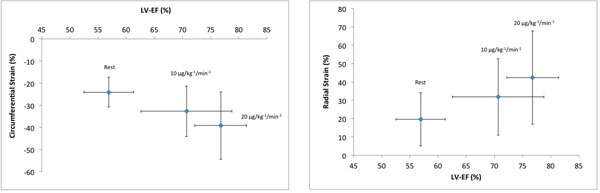
The figure shows changes in circumferential and radial strain in respect to changes of left ventricular ejection fraction (EF) at rest and with dobutamine stress (10 and 20 μg/kg-1/min-1). LV=left ventricle, EF=ejection fraction.

